# Histomorphological Characteristics and Pathological Types of Hyperproliferation of Gastric Surface Epithelial Cells

**DOI:** 10.1155/2021/8828326

**Published:** 2021-03-09

**Authors:** Yangkun Wang, Lan Shen, Guang Zhao, Baohui Li, Jianxue Bu, Chaoya Zhu, Bo Jiang, Sunan Wang

**Affiliations:** ^1^Department of Pathology, Shenzhen Hospital of Southern Medical University, Shenzhen, Guangdong Province 518100, China; ^2^Department of Pathology, The 989th Hospital of the Joint Logistics Support Force of the Chinese People's Liberation Army, Luoyang, Henan Province 471031, China; ^3^Department of Pathology, The Third Affiliated Hospital of Zhengzhou University, Zhengzhou, Henan Province 450052, China; ^4^The 990th Hospital of Joint Logistics Support Force of the Chinese People's Liberation Army, Zhumadian, Henan Province 463000, China; ^5^Shen Zhen Polytechnic, Shenzhen, Guangdong Province 518055, China

## Abstract

**Objective:**

To investigate the histomorphological characteristics and pathological types of hyperproliferation of gastric surface epithelial cells.

**Methods:**

Hematoxylin and Eosin, Periodic acid–Schiff, and immunohistochemical staining were performed on biopsy specimens obtained from 723 patients with hyperproliferation of gastric surface epithelial cells and/or hyperplasia of gastric pits. Follow-up gastroscopic reexaminations were performed on 475 patients included. Improvement probability was analyzed using Kaplan-Meyer as well as Cox proportional hazards models.

**Results:**

Seven different histomorphologies and clinicopathologies of hyperproliferation of gastric surface epithelial cells were identified: (1) common hyperplasia of gastric epithelial cells, which was characterized by focal glandular epithelial hyperplasia of gastric pits with chronic inflammation; (2) drug-induced hyperplasia of gastric epithelial cells, which was characterized by increased hyperplasia of gastric pits and cells arranged in a monolayer; (3) Helicobacter pylori (Hp) infection-induced hyperplasia of gastric epithelial cells, which was characterized by the disappearance of oval, spherical, and bounded membrane-enclosed mucus-containing granules in the cytoplasm and on the nucleus together with cytoplasmic swelling and vacuolation; (4) metaplastic hyperplasia of gastric epithelial cells, which was characterized by the coexistence of intestinal metaplastic cells with hyperplastic gastric epithelial cells; (5) atrophic hyperplasia of gastric epithelial cells, which was characterized by the mucosal atrophy accompanied with hyperplasia of gastric pits; (6) low-grade neoplasia of epithelial cells, which was characterized by the mild to moderate dysplasia of gastric epithelial cells; and (7) high-grade neoplasia of epithelial cells, which was characterized by the evident dysplasia of hyperplastic epithelial cells and losses of cell polarity. The different pathological types are associated with different improvement probabilities.

**Conclusions:**

This study demonstrated the histomorphological characteristics and pathological types, which might guide clinicians to track malignant cell transformation, perform precise treatment, predict the clinical prognosis, and control the development of gastric cancer.

## 1. Introduction

Gastric cancer remains the third leading cause of cancer-related deaths in the world [1]. The mortality from gastric cancer can be reduced because of the early-stage tumor detection and clinic pathological diagnosis [2]. Early detection of gastric cancer is increasing with the application of screening endoscopy [3]. Because gastroscopy provides the character and extent of the lesions, which provides a reference for clinicians to make accurate diagnoses and performing precise treatment [4–7]. In addition, histopathological examination is considered necessary for the clinic pathological diagnosis due to the vague and nonspecific clinical symptoms [8, 9].

The hyperproliferation of surface epithelial cells of the gastric mucosa, which secrete protective and lubricant insoluble mucus containing high concentrations of bicarbonate, is also known as hyperplasia of gastric pits and is of diagnostic value in pathohistological practice [10]. Because this epithelial cell proliferation, which breaks the balance between cell proliferation and apoptosis, is accepted as one of the risk factors for gastric carcinogenesis [11]. Malignancies can develop through benign hyperplasia of gastric surface epithelial cells and are preceded by atypical hyperplasia, which can progress into intraepithelial neoplasia and malignant forms [11, 12]. The proliferation of surface epithelial cells of the gastric mucosa reportedly results from infected gastric mucosa, drug stimulation, immune factors and genetic factors, etc. In addition to many diverse etiologies, there are various histomorphologies caused by the proliferation of gastric epithelial cells, which lead to great differences in treatment and prognosis [13]. Therefore, detecting the histomorphologies caused by hyperplasia of gastric surface epithelial cells is significantly important for clinicians to understand the development process of lesions, thus making accurate diagnosis and performing precise treatment.

In the present study, 723 cases of hyperplasia of gastric pits and/or hyperplasia of gastric surface epithelial cells in gastroscopic biopsy specimens were collected, of which 475 cases were reexamined by gastroscopic biopsy, to investigate the morphological and pathological characteristics caused by hyperplasia of gastric surface epithelial cells. This study is conducive for clinicians to perform accurate treatment of gastric surface epithelial cell hyperplasia/gastric pits hyperplasia and the follow-up of malignant transformation. In addition, our findings provide insight into the nature and development process of lesions.

## 2. Materials and Methods

### 2.1. Study Subjects

A total of 723 patients diagnosed with hyperplasia of gastric pits and/or hyperplasia of gastric surface epithelial cells were included in this study. These 723 patients included 448 men and 275 women were hospitalized in the department of gastroenterology of Shenzhen Hospital of Southern Medical University, the 990th Hospital of Joint Logistics Support force of the Chinese People's Liberation Army, and the Third Affiliated Hospital of Zhengzhou University, and underwent endoscopic examination between December 2018 and December 2019. The age of patients ranged from 24 to 78 years, with an average age of 45.2 years. Two or three tissues were harvested from each site of a patient and were used as gastroscopic biopsy specimens. This study was approved by the ethics committee of Shenzhen Hospital of Southern Medical University, the 990th Hospital of Joint Logistics Support force of the Chinese People's Liberation Army, and the Third Affiliated Hospital of Zhengzhou University. Written informed consent was obtained from all study subjects prior to the procedure.

### 2.2. Materials

The Hematoxylin and Eosin (H&E) staining Kit, Periodic Acid Schiff (PAS) Stain Kit, and Envision Kit were obtained from Shenzhen Dameng Bio-Medical Technology Co., Ltd. All primary antibodies were obtained from Shenzhen Dameng Bio-Medical Technology Co., Ltd. Secondary antibody for immunohistochemistry and DAB (3,3′-diaminobenzidine) chromogen kit were bought from Shenzhen Dameng Bio-Medical Technology Co., Ltd.

### 2.3. Hematoxylin and Eosin (H&E) Staining

Biopsies were fixed in neutral buffered 10% formalin (to preserve their histological structure), embedded in paraffin, and sectioned at four micrometer. Next, sections were stained with H&E according to the following protocol. The sections were deparaffinized in xylene for 15 min twice, gradually hydrated in 100%, 80%, and 70% alcohol for 5 min twice. After being washed in distilled water for 5 min, the sections were immersed in Hematoxylin solution for 5 min and then dipped in distilled water for 2-3 s. Next, sections were differentiated in 1% HCl ethanol for 1-3 s and rinsed with distilled water for 30 s. After being washed in distilled water for 30 s, the sections were immersed in 80%, 90%, and 100% alcohol for 10 min. The sections were subsequently incubated in 0.5% eosin aqueous solution for 3 min. The gastric tissue structure and cell morphology were observed under the optical microscope.

### 2.4. Periodic Acid–Schiff (PAS) Staining

Biopsies were fixed in neutral buffered 10% formalin, embedded in paraffin, and cut into 4 *μ*m thick sections. Next, gastric tissue sections were stained with PAS according to the following protocol. The sections were deparaffinized in xylene and graded alcohols. After being washed in distilled water, the sections were immersed into periodic acid solution for 15-20 min, followed by being washed with distilled water. The sections were then treated with Schiff's reagent for 30-60 min and rinsed with sulphurous acid solution. Sections were subsequently washed with distilled water for 2-3 min and counterstained with methylgreen for 10-20 min. After staining, tissue sections were differentiated with hydrochloric acid alcohol, clarified in xylol, and mounted. Positive cells (red; nuclei, blue) were monitored using the optical microscope.

### 2.5. EnVision Two-Step Immunohistochemical Staining

Immunohistochemical staining was performed with an Envision Kit. The operations are performed according to the manufacturer's instructions. Briefly, paraffin-embedded gastric tissue sections were deparaffinized, hydrated, and rinsed with distilled water. Then, the sections were placed in Tris-buffered saline (TBS) for 10 min. Next, endogenous peroxidase activity was blocked by incubating sections for 5 min in peroxidase blocking reagent containing H_2_O_2_ and NaN_3_. Sections were subsequently treated with TBS for 10 min. Each of the primary antibodies (CEA, CK7, CK20, Hp, MUC1, MUC2, MUC5AC, MUC6, p53, and Ki-67) was incubated with the sections for 30 min at room temperature. After a 10-min wash in TBS, sections were incubated in EnVisionTM. After a 10-min wash in TBS, a secondary antibody was applied for 10 min. The chromogenic substrate solution was incubated for 10 min followed by distilled water rinsing. Color was developed with DAB and sections were counterstained with hematoxylin. Known gastric mucosa sections were used as positive controls, and PBS buffer instead of the primary antibody was used as the negative control. The positive expression of MUC2, MUC5AC, and MUC6 was determined when yellow-brown granules were found in the cytoplasm. The positive expression of P53 and Ki-67 was determined when yellow-brown granules were observed within the cell nucleus.

### 2.6. Follow-Up Examination

The follow-up was performed on 475 patients who were pathologically diagnosed as hyperproliferation of gastric surface epithelial cells and/or hyperplasia of gastric pits. At least two tissues were harvested from each site of the patient between December 2018 and December 2019. The follow-up included endoscopic, histomorphological, and immunohistochemical examinations and was performed from December 2018 to June 2020. The time range for reexamination was divided into 3 stages: 1^st^~3^rd^ months, 4^th^~6^th^ months, and 7 ^th^~12^th^ months.

### 2.7. Statistics

Cox proportional hazards models were used to calculate the hazard ratios (HRs) and 95% confidence intervals (CI). *p* values of 0.05 were considered significant.

## 3. Results

### 3.1. The Formation of Epithelial Cell Lesion on Gastric Mucosal Surface

**Figure 1:**
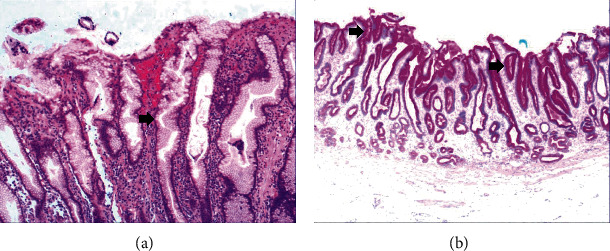
The histomorphologic characteristics of common hyperplasia of gastric epithelial cells. (a) H&E staining results showing morphologically focal glandular epithelial hyperplasia, with chronic inflammation of gastric mucosa, with the height of hyperplastic gastric pits between 0.5 and 1.0 mm. Characteristic image at 200x objective magnification was shown. (b) PAS staining results showing gastric epithelial cells with strong expression of neutral mucin. Characteristic image at 100x objective magnification was shown. The arrows refer to hyperplastic gastric pits.

To investigate the histomorphologic changes caused by hyperplasia of gastric epithelial cells, we performed H&E staining and immunohistochemical staining on gastroscopic biopsies. Results from H&E staining showed that commonly the hyperplasia of gastric mucosal epithelium is morphologically focal glandular epithelial hyperplasia, and the height of hyperplastic gastric pits is between 0.5 and 1.0 mm when there is lymphocyte infiltration (Figure 1(a)). The hyperplasia of gastric pits indicated the histomorphological hyperplasia of the surface epithelium and gastric foveolar epithelium.

**Figure 2:**
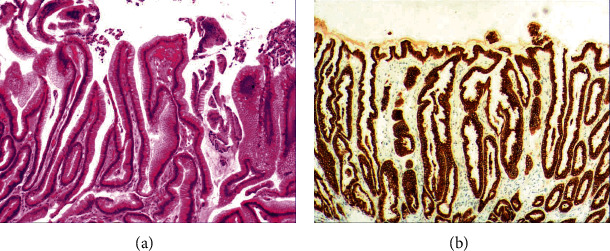
The histomorphologic characteristics of drug-induced hyperplasia of gastric epithelial cells. (a) H&E results showing no or very little inflammatory cell infiltration in the stroma, with the height of hyperplastic gastric epithelial cells between 1 and 1.5 mm and cells arranged in a monolayer. (b) Immunohistochemical staining results showing positive expression of MUC5AC. Characteristic images of each group at 200x objective magnification were shown.

**Figure 3:**
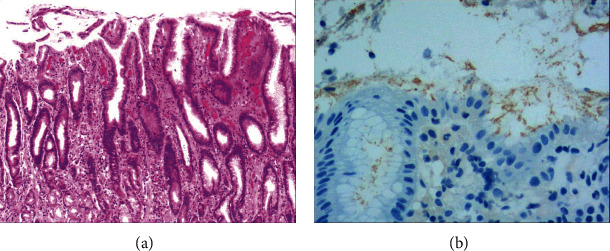
The histomorphologic characteristics of Hp infection-induced hyperplasia of gastric epithelial cells. (a) H&E staining results showing the disappearance of oval, spherical, and bounded membrane-enclosed mucus-containing granules in the cytoplasm and on the nucleus are disappeared, with cytoplasmic swelling and vacuolation. Characteristic image at 100x objective magnification was shown. (b) Immunohistochemical staining results showing positive expression of Hp. Characteristic image at 400x objective magnification was shown.

While the drug-induced hyperplasia of gastric epithelial cells is characterized by no or very little inflammatory cell infiltration in the stroma, with the height of hyperplastic surface epithelial cells between 1 and 1.5 mm and the cells being arranged in a monolayer (Figure 2(a)).  Figure 3(a) shows that oval, spherical, and bounded membrane-enclosed mucus-containing granules in the cytoplasm and on the nucleus are disappeared, with cytoplasmic swelling and vacuolation.  Figure 3(a) also demonstrates that hyperplastic gastric pits are histologically thick and wide, with wide interstitium caused by inflammation, edema, and vascular congestion.  Figure 3(b) shows a positive expression of Hp, confirming the Hp infection. These findings confirmed that the damage to surface epithelial cells caused by Hp, drug stimulation, and autoimmune factors can lead to the hyperplasia of gastric epithelial cells. It was also found that hyperplasia beyond the normal range caused hyperplasia of gastric pits, with the height of hyperplastic gastric pits exceeding twice of the height of normal gastric pits, and more than three consecutive pits proliferated.

**Figure 4:**
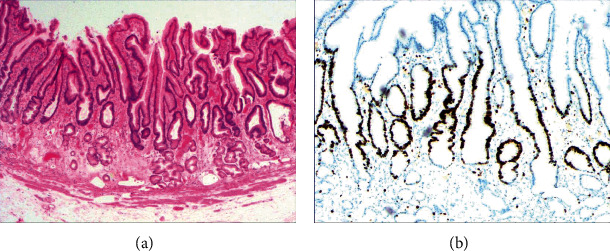
The histomorphologic characteristics of low-grade neoplasia of epithelial cells. (a) H&E staining results showing mild to moderate dysplasia of gastric epithelial cells, which were located at the base of the glandular epithelium, with increased nuclear length, retained polarity, and visible mitoses. Characteristic image at 100x objective magnification was shown. (b) Immunohistochemical staining results showing the expression of ki-67. Characteristic image at 200x objective magnification was shown.

**Figure 5:**
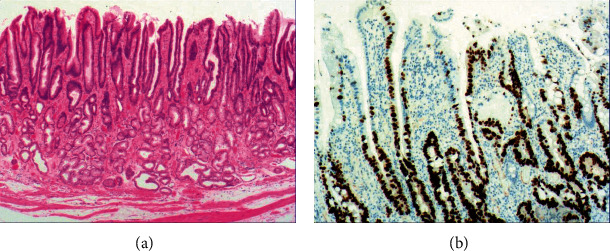
The histomorphologic characteristics of high-grade neoplasia of epithelial cells. (a) H&E staining results showing evident dysplasia of hyperplastic epithelial cells, with cells morphologically columnar to cuboidal, large nuclei, increased nuclear cytoplasmic ratio, prominent nucleoli, and increased mitotic figures. Characteristic image at 100x objective magnification was shown. (b) Immunohistochemical staining results showing the expression of p53. Characteristic image at 200x objective magnification was shown.

Figure 4(a) shows mild to moderate dysplasia of gastric epithelial cells which were located at the base of the glandular epithelium, with increased nuclear length, retained polarity, and visible mitoses.  Figure 5(a) demonstrates evident dysplasia of hyperplastic epithelial cells and cell polarity disorder. Besides, cells were morphologically columnar to cuboidal, with large nuclei, increased nuclear cytoplasmic ratio, prominent nucleoli, and increased mitotic figures (Figure 5(a)). These varying degrees of cellular and structural atypias suggested hyperplasia and dysplasia of gastric epithelial cells. These observations also indicated the neoplastic hyperplasia caused by hyperplasia and dysplasia of gastric epithelial cells. In this study, the hyperplasia and dysplasia of gastric epithelial cells, which are characterized by varying degrees of cellular and structural atypias and are able to result in neoplastic hyperplasia, are referred to as the “epithelial cell lesion on gastric mucosal surface.” The positive PAS staining (indicating neutral mucin) showed in Figure 1 suggested the presence of gastric epithelial cells accompanied with hyperplasia of gastric pits. Therefore, “epithelial cell lesion on gastric mucosal surface” is also referred to as the “gastric pits lesions or columnar mucous cell lesions”.

### 3.2. Histomorphologic Characteristics of Epithelial Cell Lesion on Gastric Mucosal Surface

**Figure 6:**
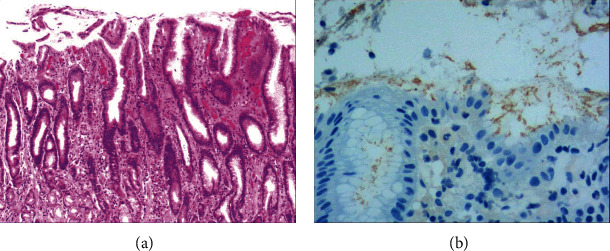
The histomorphologic characteristics of metaplastic hyperplasia of gastric epithelial cells. (a) H&E staining results showing coexistence of intestinal metaplastic cells with hyperplastic gastric epithelial cells, with cells organized in a monolayer or stratified epithelium arrangement and a nuclear length 1–2 times of that in normal epithelial cells. Characteristic image at 100x objective magnification was shown. (b) Immunohistochemical staining results showing positive expression of MUC2. Characteristic image at 200x objective magnification was shown.

**Figure 7:**
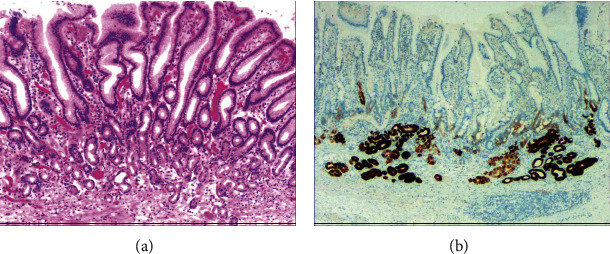
The histomorphologic characteristics of atrophic hyperplasia of gastric epithelial cells. (a) H&E staining results showing mucosal atrophy (loss of glands) accompanied with hyperplasia of gastric pits, with very few atrophic pyloric glands. (b) Immunohistochemical staining results showing positive expression of MUC6 in pyloric glands. Characteristic images at 100x objective magnification were shown.

As shown in Figure 1(a), the common hyperplasia of gastric epithelial cells is morphologically characterized by focal glandular epithelial hyperplasia, accompanied by chronic inflammation of gastric mucosa. The height of hyperplastic gastric pits is between 0.5 and 1.0 mm. (Figure 1(a)). While the drug-induced hyperplasia of gastric epithelial cells is characterized by no or very little inflammatory cell infiltration in the stroma, with the cells arranged in a monolayer (Figure 2(a)). Infection-induced hyperplasia of gastric epithelial cells is characterized by cytoplasmic swelling and vacuolation. As shown in Figure 3(a), oval, spherical, and bounded membrane-enclosed mucus-containing granules in the cytoplasm and on the nucleus are disappeared, with cytoplasmic swelling and vacuolation.  Figure 3(a) also demonstrates that hyperplastic gastric pits are histologically thick and wide, with wide interstitium caused by inflammation, edema, and vascular congestion.  Figure 6(a) shows that intestinal metaplastic cells coexisted with hyperplastic gastric epithelial cells, and cells were organized in a monolayer or stratified epithelium arrangement, with a nuclear length 1–2 times of that in normal epithelial cells. In this study, such a lesion is referred to as “metaplastic hyperplasia of gastric epithelial cells.” Figure 7(a) shows mucosal atrophy (loss of glands) accompanied with hyperplasia of gastric pits, with varying degrees of decrease and even disappearance of gastric fundus glands, cardiac glands, and pyloric glands. However, such hyperplasia is compensatory and regional and cannot be determined morphologically as intraepithelial neoplasia. In this study, such a lesion is called “atrophic epithelial hyperplasia”. Figure 4(a) shows histological morphology characterized by mild to moderate dysplasia of gastric epithelial cells, which were located at the base of the glandular epithelium, with increased nuclear length and retained polarity. Such hyperplasia is referred to as “low-grade neoplasia of epithelial cells.” Figure 5(a) demonstrates evident dysplasia of hyperplastic epithelial cells. Besides, cells were morphologically columnar to cuboidal, with large nuclei, increased nuclear cytoplasmic ratio, prominent nucleoli, and increased mitotic figures (Figure 5(a)). In this study, such a lesion is referred to as “high-grade neoplasia of epithelial cells.” The pathological types and histological diagnostic criteria of epithelial cell lesions on gastric mucosal surface are explicated in Table 1.

### 3.3. Results from PAS Staining and Immunohistochemical Staining

To identify the hyperplasia of gastric epithelial cells, PAS staining was performed. In addition, to investigate the expression of MUC1, MUC2 [14], MUC5AC (a gastric-type secreted mucin), MUC6, CK7, CK20, CEA, p53, and Ki-67 in the development of epithelial cell lesions on gastric mucosal surface, immunohistochemical staining was performed. The PAS staining and immunohistochemical staining results are explicated in Table 2. According to Table 2, positive expression of Hp was only identified in the case of Hp infection-induced hyperplasia of gastric epithelial cells, which was also confirmed by PAS staining (Figure 3(b)). CK7 and CK20 were positively expressed (+) in cases of low-grade neoplasia of epithelial cells and high-grade neoplasia of epithelial cells, while negatively expressed in other cases (Table 2). Negative expression of CEA was detected in cases of common and drug-induced hyperplasia of gastric epithelial cells, while positive expression (+) of CEA was identified in cases of Hp infection-induced, metaplastic and atrophic hyperplasia of gastric epithelial cells. The strongly positive expression (+ + +) of CEA was found in cases of low-grade and high-grade neoplasia of epithelial cells. Negative expression of MUC1 was found in cases of the common, drug-induced, and Hp infection-induced hyperplasia of gastric epithelial cells, while positive expression (+) of MUC1 was identified in cases of metaplastic hyperplasia of gastric epithelial cells, atrophic hyperplasia of gastric epithelial cells, low-grade neoplasia of epithelial cells, and high-grade neoplasia of epithelial cells. The positive expression of MUC2 was only identified in the case of metaplastic hyperplasia of gastric epithelial cells, which was confirmed by PAS staining (Figure 6(b)). Strongly positive expression of MUC5AC was identified in cases of common and drug-induced hyperplasia of gastric epithelial cells, which was confirmed by PAS staining (Figure 2(b)). Additionally, MUC5AC was found to positively be expressed in the cases of Hp-induced, metaplastic, and atrophic hyperplasia of gastric epithelial cells while negatively expressed in cases of low-grade and high-grade neoplasia of epithelial cells. Negative expression of MUC6 was found in cases of the common, drug-induced, and Hp infection-induced hyperplasia of gastric epithelial cells, while positive expression (+) of MUC6 was identified in cases of atrophic hyperplasia of gastric epithelial cells, low-grade neoplasia of epithelial cells, and high-grade neoplasia of epithelial cells. The strongly positive expression (+) of MUC6 was identified in cases of metaplastic hyperplasia of gastric epithelial cells. It was also found that P53 showed positive expression in the cases of low-grade neoplasia of epithelial cells and strongly positive in the cases of high-grade neoplasia of epithelial cells, which was also confirmed by PAS staining (Figure 5(b)). The percentage of Ki67-positive cells in cases of the common, drug-induced, Hp infection-induced, metaplastic, atrophic hyperplasia, and low-grade and high-grade neoplasia of epithelial cells was 1~7%, 11~19%, 14~21%, 9~32%, 18~34%, 26~35%, and 32~40%, respectively (Table 2).

### 3.4. Results from Follow-Up Examination

Follow-up examination was performed on cases with at least two harvested tissues. Therefore, a total of 475 patients were included in this study. Follow-up findings were shown in Table 3. Of the 475 patients, 214 patients (45.1%) were cases of common hyperplasia of gastric epithelial cells, with 74.3% improved and cured cases while 6.5% worsened cases. The 475 patients included 22 cases of drug-induced hyperplasia of gastric epithelial cells, with the improved and cured cases of 90.0%. The 475 patients also included 115 cases of Hp-induced hyperplasia of gastric epithelial cells, with the improved and cured cases of 73.9% and worsened cases of 10.9%. There were 37 cases of metaplastic hyperplasia of gastric epithelial cells, with the improved and cured cases of 51.4% and worsened cases of 13.5%. 29 cases of low-grade neoplasia of epithelial cells were followed up, and only 17.2% were found to be improved and cured cases while 10.3% were found to be worsened. These findings indicated that metaplastic hyperplasia of gastric epithelial cells, atrophic hyperplasia of gastric epithelial cells, and low-grade neoplasia of epithelial cells showed high rate of malignant transformation despite morphologically benign lesions.

### 3.5. Results from Univariate and Multivariate Models

**Figure 8:**
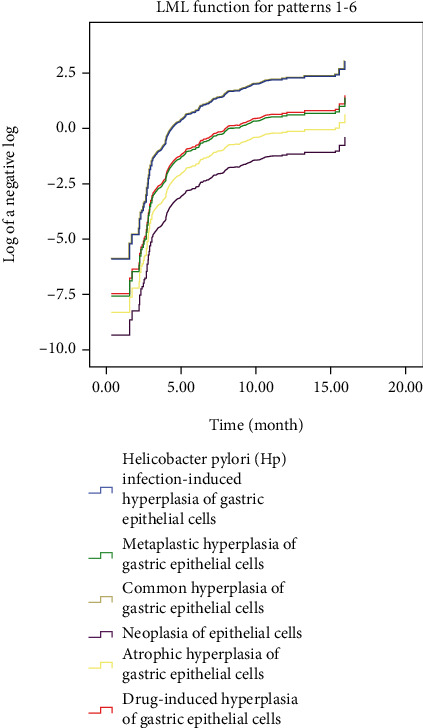
The Kaplan-Meyer survival plots of the study population.

Kaplan-Meier survival curves (Figure 8) demonstrates that the proportionality assumption was always satisfied. Next, Cox proportional hazards model (or Cox regression) was used to evaluate the probability of cure and improvement. The Cox model shows that with drug-induced hyperplasia of gastric epithelial cells used as a reference, common hyperplasia of gastric epithelial cells, and Hp infection-induced hyperplasia of gastric epithelial cells were associated with higher improvement probability [HR: 4.95 (95% CI: 2.95–8.30), *p* < 0.001 and HR: 4.81 (95% CI: 2.81–8.21), *p* < 0.001, respectively] (Table 4). The Cox model also demonstrates reduced improvement probability related with atrophic hyperplasia of gastric epithelial cells [HR: 0.43 (95% CI: 0.23–0.82), *p* = 0.01] and neoplasia of epithelial cells [HR: 0.16 (95% CI: 0.04–0.67), *p* = 0.01] (Table 4). These results are consistent with the results from follow-up examination, thus indicating that the histomorphological characteristics and pathological types are helpful to predict the clinical prognosis of the hyperplasia and dysplasia of gastric epithelial cells.

## 4. Discussion

Normally, the gastric mucosa is histologically composed of epithelium, lamina propria, and mucosa. Gastric epithelial cells are also known as surface epithelial cells or gastric foveolar epithelial cells. Most gastric mucosal surface epithelial cells are surface mucous cells, and cells are arranged in a monolayer, with an inconspicuous nucleolus. There are oval, spherical, and bounded membrane-enclosed mucus-containing granules in the cytoplasm and on the nucleus with strongly positive PAS staining [15]. Studies have shown that the drug stimulation, autoimmune diseases, and infection (especially the Hp infection) are able to destroy the structure of gastric epithelial cells, thus leading to hyperplasia of gastric mucosal surface epithelial cells [16, 17]. In this study, the hyperplasia and dysplasia of gastric epithelial cells are referred to as the “epithelial cell lesion on gastric mucosal surface.” Many etiologies can result in the hyperplasia of gastric epithelial cells, which also show different histomorphological characteristics with the development of lesions. However, gastric foveolar hyperplasia described in the clinicopathological case report reflects few histomorphological features, thus providing clinicians little valuable information to assess the relationship between gastric foveolar hyperplasia and carcinogenesis. The present study provides the histomorphological features and pathological types of “epithelial cell lesion on gastric mucosal surface,” which is of importance for clinicians to track malignant cell transformation, perform precise treatment, predict the clinical prognosis, and control the occurrence and development of gastric cancer.

Gastric mucosal atrophy is accepted as an important precancerous lesion [18]. Worsened cases (6.5%) of common hyperplasia of gastric epithelial cells suggest that common hyperplasia of gastric epithelial cells is morphologically benign lesions with potential to be advanced. 90.9% of cases of drug-induced hyperplasia of gastric epithelial cells were found to be improved and cured, suggesting histologically benign lesions with a low probability of malignant transformation, which indicates that clinicians can make the patients recover by adjusting the drug dose. Gastroscopic biopsy reexamination is recommended within half a year for patients with drug-induced hyperplasia of gastric epithelial cells. The present study demonstrated that 7.0% cases of drug-induced hyperplasia of gastric epithelial cells were exacerbated, while 73.9% cases of drug-induced hyperplasia of gastric epithelial cells were improved and cured, which indicates the vast majority of cured cases after aggressive anti-Hp therapy. Semiannual gastroscopic biopsy reexaminations are recommended to closely monitor the proliferation of epithelial cells because Hp infection has been recognized as the main cause of gastric adenocarcinoma [17, 19]. 10.9% cases of metaplastic hyperplasia of gastric epithelial cells were found to be exacerbated in our work, which indicates a high tendency to malignant transformation. Intestinal metaplasia is reported as a defensive, reparative, and reactive response to inflammatory stimuli and injury, and hyperplasia no longer continues once the cause of hyperplasia is eliminated [20, 21]. Therefore, semiannual gastroscopic biopsy reexamination is also recommended for patients with metaplastic hyperplasia of gastric epithelial cells. In the present study, 10.3% cases of low-grade epithelial neoplasia were aggravated. Consistent with previous studies, closely followed up is recommended for patients with low-grade epithelial neoplasia [22]. We recommend semiannual gastroscopic biopsy reexaminations for patients with low-grade epithelial neoplasia and suggest endoscopic submucosal dissection (ESD) for lesion resection once aggravated morphological changes are observed. Current treatment strategies for high-grade intraepithelial neoplasia include endoscopic therapy, surgical treatment, and follow-up [23]. In the present study, 12 patients were with high-grade neoplasia of epithelial cells. These 12 cases were followed up with endoscopic reexamination within 3 months after treatment. Patients with negative results were reexamined endoscopically within 6 months after treatment. Patients with negative results from reexamination were reexamined endoscopically 1 year later. Patients with negative results for two consecutive times were reexamined endoscopically every 1 year. Our findings suggest that the classification of epithelial cell lesions on gastric mucosal surface according to the morphological features, etiologies, and development rules is conducive for clinicians to achieve precise treatment. Consistently, Cox model showed increased improvement probability in cases of common hyperplasia of gastric epithelial cells and Hp infection-induced hyperplasia of gastric epithelial cells [HR: 4.95 (95% CI: 2.95–8.30), *p* < 0.001 and HR: 4.81 (95% CI: 2.81–8.21), *p* < 0.001, respectively] (Table 4) while reduced improvement probability in cases of atrophic hyperplasia of gastric epithelial cells [HR: 0.43 (95% CI: 0.23–0.82), *p* = 0.01] and neoplasia of epithelial cells [HR: 0.16 (95% CI: 0.04–0.67), *p* = 0.01] (Table 4). These results indicate that the histomorphological characteristics and pathological types are helpful to predict the clinical prognosis of the hyperplasia and dysplasia of gastric epithelial cells.

The histomorphological characteristics and clinical outcomes of the disease are strongly associated with its biological characteristics. In the present study, increased expression of Ki67 was observed in the cases of drug-induced (11~19%), Hp infection-induced (14~21%), metaplastic (9~32%), atrophic hyperplasia (18~34%), low-grade neoplasia (26~35%), and high-grade neoplasia (32~40%) (Table 2). These data suggest the increased cell proliferation and tendency towards carcinogenesis. CK20 expression is accepted to be related to tumor invasion and metastasis. Consistently, in the present study, CK20 is found to be positively expressed (+) in the cases of low-grade neoplasia of epithelial cells and high-grade neoplasia of epithelial cells, while negatively expressed in other cases (Table 2) [24]. Prerequisite of tumor growth is the rapid blood vessel formation (angiogenesis) to support nutrients and oxygen for highly proliferating tumor cells [14]. Angiogenesis is strongly associated to the tumor invasion and metastasis [25]. Therefore, further investigation of tumor vessel formation and its mechanisms contributes to the development of new strategies to treat cancer. Vascular endothelial growth factors (VEGFs) family, epidermal growth factor (EGF) family, resistin-like molecule-*α* (RELM-*α*), platelet-derived growth factor-*β*, hypoxia-inducible factors, and microRNAs (miRNAs) are accepted to be involved in the induction and progression of angiogenesis [26]. microRNAs (miRNAs) (e.g., miR-135a; miR-377; miR-218, miR-130, miR-495) are accepted to be critical regulators of tumor angiogenesis and received focus as promising targets in new antiangiogenic therapies [14]. Therefore, further investigation will focus on the miRNAs expression in the seven cases of gastric surface epithelial cells described in the current study, which would contribute to the tissue-specific delivery of miRNAs for individualized treatment of the disease.

Consistent with previous studies [27], CK7, CK20, CEA, P53, and Ki-67 showed increased expression in cases of low-grade intraepithelial neoplasia and high-grade intraepithelial neoplasia. These findings suggest that combined detection of CK7, CK20, CEA, P53, and Ki-67 is an important indicator of intraepithelial neoplasia. Our results showed that MUC5AC expression was decreased in the gastric foveolar epithelium of Hp-infected patients, which is in agreement with that reported by Tanaka et al. [28] and Byrd et al. [29] while in contrast to that reported by Teixeira et al. [30]. MUC5AC was found to be negatively expressed in cases of low-grade neoplasia of epithelial cells and high-grade neoplasia of epithelial cells, indicating that MUC5AC can be used as a marker for malignant transformation of gastric mucosal epithelium. MUC2 has been reported as an intestinal epithelial marker [12]. In the current study, positive (+) expression of MUC2 was only reported in cases of metaplastic hyperplasia of gastric epithelial cells. These findings indicate that combined staining for MUC2 and MUC5AC can be used to detect the proliferation and transformation of gastric mucosal surface epithelial cells. Consistent with previous studies [31], strong positive MUC6 expression, in addition to the expression of MUC2, was observed in cases of metaplastic hyperplasia. The systematical evaluation of protein expression in this study would provide clinicians the in-depth understanding of the development process of hyperplasia.

New clinical treatment concepts result from high-quality clinical evidence, which often comes from randomized controlled studies. However, randomized controlled studies are limited by high cost, long time, and difficulty in enrollment clinically [32]. Fortunately, the study based on retrospective database is close to the realistic clinical practice, covers a large sample sizes, and has high feasibility [33]. In the present retrospective study, the precious clinical data of more than 700 cases in multiple centers were collected as a remedy for alternative randomized controlled studies. Based on the retrospective database, seven different histomorphologies and clinicopathologies of hyperproliferation of gastric surface epithelial cells were identified. Our findings might provide guidance to select the treatment measures according to the actual condition and wishes of patients. Despite these encouraging results, limitations exist in the current study. Because of this retrospective, population-based cohort study, further experiments are needed to confirm the conclusions drawn from this study.

## 5. Conclusions

In summary, epithelial cell lesions on gastric mucosal surface are the result of a variety of different factors. Currently, hyperplasia of gastric pits described in the clinicopathological report cannot reflect the degree of gastric mucosal injury, thus not conducive to guide clinicians to achieve accurate treatment. The present study demonstrated the histomorphological characteristics and pathological types of epithelial cell lesions on gastric mucosal surface, as well as the changes in expression of Hp, MUC1, MUC2, MUC5AC, MUC6, CK7, CK20, CEA, p53, and Ki-67 during the development of the lesions. Our findings provide in-depth understanding of the nature and the development of epithelial cell lesions on gastric mucosal surface, which is conducive to clinicians to perform follow-up of malignant cell transformation, predict the clinical prognosis, and achieve accurate treatment to control the occurrence and development of gastric cancer.

## Figures and Tables

**Table 1:** Histomorphologic changes caused by hyperplasia of gastric epithelial cells.

Hyperplasia types	Histomorphologic characteristics
Common hyperplasia of gastric epithelial cells	Morphologically focal glandular epithelial hyperplasia, with chronic inflammation of gastric mucosa; the height of hyperplastic gastric pits between 0.5 and 1.0 mm.
Drug-induced hyperplasia of gastric epithelial cells	No or very little inflammatory cell infiltration in the stroma; the height of hyperplastic gastric pits is between 1 and 1.5 mm, with the cells arranged in a monolayer.
Hp infection-induced hyperplasia of gastric epithelial cells	Oval, spherical, and bounded membrane-enclosed mucus-containing granules in the cytoplasm and on the nucleus are disappeared, with cytoplasmic swelling and vacuolation and positive expression of Hp
Metaplastic hyperplasia of gastric epithelial cells	Coexistence of intestinal metaplastic cells with hyperplastic gastric epithelial cells; cells were organized in a monolayer or stratified epithelium arrangement, with a nuclear length 1–2 times of that in normal epithelial cells
Atrophic hyperplasia of gastric epithelial cells	Compensatory and regional hyperplasia; it cannot be determined morphologically as intraepithelial neoplasia; mucosal atrophy (loss of glands) accompanied with hyperplasia of gastric pits, with varying degrees of decrease and even disappearance of gastric fundus glands, cardiac glands, and pyloric glands.
Low-grade neoplasia of epithelial cells	Mild to moderate dysplasia of gastric epithelial cells, which were located at the base of the glandular epithelium, with increased nuclear length, retained polarity, and visible mitoses.
High-grade neoplasia of epithelial cells	Evident dysplasia of hyperplastic epithelial cells; cells were morphologically columnar to cuboidal, with large nuclei, increased nuclear cytoplasmic ratio, prominent nucleoli, and increased mitotic figures.

**Table 2:** PAS staining and immunohistochemical staining results.

Hyperplasia types	Hp	CK7	CK20	CEA	MUC1	MUC2	MUC5AC	MUC6	p53	PAS	Percentage of Ki67-positive cells
Common hyperplasia of gastric epithelial cells	—	—	—	—	—	—	+++	—	—	+	1~7%
Drug-induced hyperplasia of gastric epithelial cells	—	—	—	—	—	—	+++	—		+	11~19%
Hp infection-induced hyperplasia of gastric epithelial cells	+	—	—	+	—	—	+	—		+	14~21%
Metaplastic hyperplasia of gastric epithelial cells	—	—	—	+	+	+	+	+++		+	9~32%
Atrophic hyperplasia of gastric epithelial cells	—	—		+	+	—	+	+		+	18~34%
Low-grade neoplasia of epithelial cells	—	+	+	+++	+	—	—	+	+	—	26~35%
High-grade neoplasia of epithelial cells	—	+	+	+++	+	—	—	+	+++	—	32~40%

**Table 3:** Follow-up results.

Hyperplasia types	Cases	Cases with little change (%)	Cured/improved cases (%)	Worsened cases (%)
Common hyperplasia of gastric epithelial cells	214	41 (19.2)	159 (74.3)	14 (6.5)
Drug-induced hyperplasia of gastric epithelial cells	22	2 (9.1)	20 (90.9)	0 (0)
Hp infection-induced hyperplasia of gastric epithelial cells	115	20 (17.4)	87 (75.7)	8 (6.9)
Metaplastic hyperplasia of gastric epithelial cells	46	14 (30.4)	27 (58.7)	5 (10.9)
Atrophic hyperplasia of gastric epithelial cells	37	9 (24.3)	23 (62.2)	5 (13.5)
Low-grade neoplasia of epithelial cells	29	21 (72.4)	5 (17.2)	3 (10.3)
High-grade neoplasia of epithelial cells	12	0	12 (100%)^∗^	0

^∗^: Lesions were all removed through ESD.

**Table 4:** Cox-modelled hazard ratio (HR) for all outcomes.

Hyperplasia types	HR	95% CI	*p*
Drug-induced hyperplasia of gastric epithelial cells	1.00	—	—
Common hyperplasia of gastric epithelial cells	4.95	2.95–8.30	<0.001
Hp infection-induced hyperplasia of gastric epithelial cells	4.81	2.81–8.21	<0.001
Metaplastic hyperplasia of gastric epithelial cells	0.90	0.48–1.68	0.74
Atrophic hyperplasia of gastric epithelial cells	0.43	0.23–0.82	0.01
Neoplasia of epithelial cells	0.16	0.04–0.67	0.01

## Data Availability

Corresponding authors and first authors are in charge of the underlying data supporting the results of this study.
