# Correction to “Histomorphological Characteristics and Pathological Types of Hyperproliferation of Gastric Surface Epithelial Cells”

**DOI:** 10.1155/grp/9831902

**Published:** 2025-12-13

Y. Wang, L. Shen, G. Zhao, B. Li, J. Bu, C. Zhu, B. Jiang, and S. Wang, “Histomorphological Characteristics and Pathological Types of Hyperproliferation of Gastric Surface Epithelial Cells,” *Gastroenterology Research and Practice* 2021, no. 1 (2021), https://doi.org/10.1155/2021/8828326.

In the article titled “Histomorphological Characteristics and Pathological Types of Hyperproliferation of Gastric Surface Epithelial Cells,” there was an error in Figure 6 related to an accidental duplication of the images from Figure 3.

The associated figure legend was unaffected, and the authors confirm that the results, discussion, and conclusion remain unaffected.

The corrected figure is shown below and is listed as Figure 6.

Figure 6The histomorphologic characteristics of metaplastic hyperplasia of gastric epithelial cells. (a) H&E staining results showing coexistence of intestinal metaplastic cells with hyperplastic gastric epithelial cells, with cells organized in a monolayer or stratified epithelium arrangement and a nuclear length 1–2 times of that in normal epithelial cells. Characteristic image at 100× objective magnification was shown. (b) Immunohistochemical staining results showing positive expression of MUC2. Characteristic image at 200× objective magnification was shown.

**Figure:**
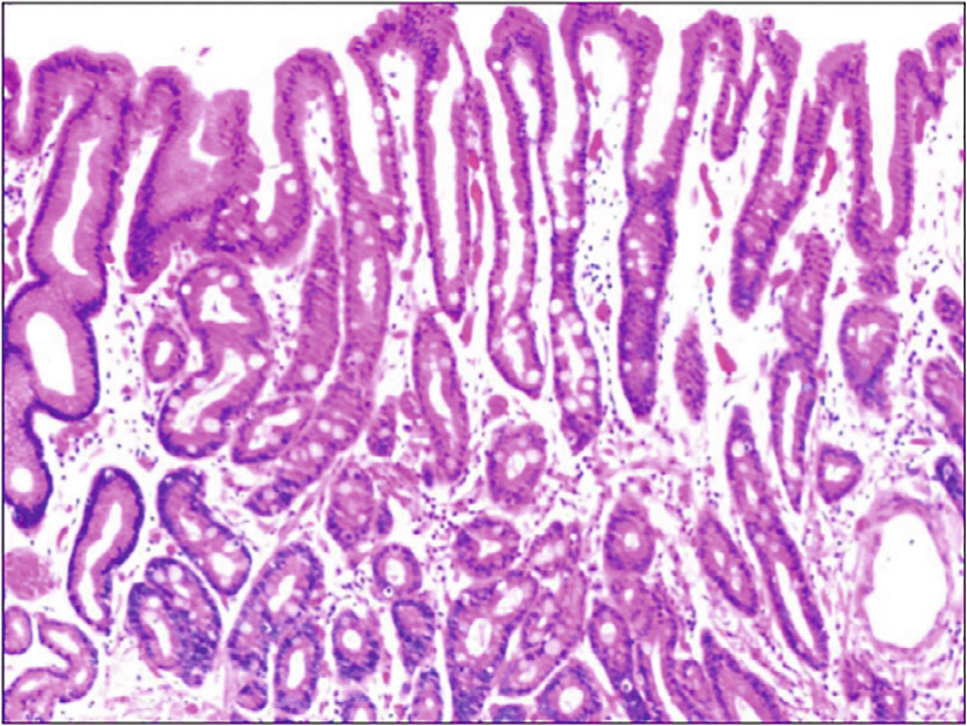
(a)

**Figure:**
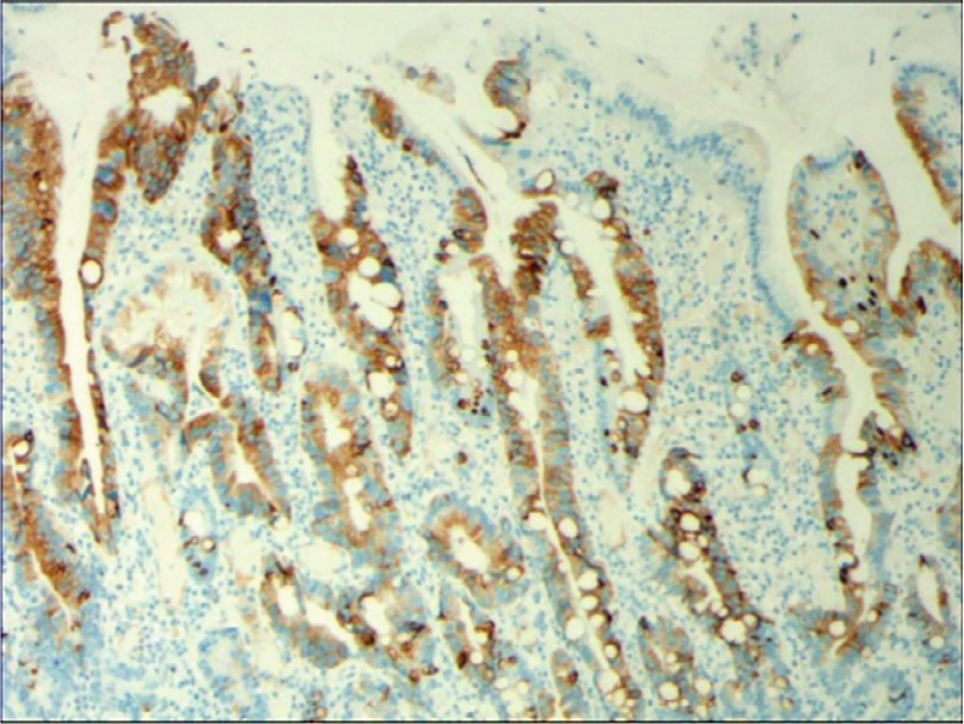
(b)

We apologize for this error.

